# The impact of COVID-19 on psychoactive products consumers

**DOI:** 10.1192/j.eurpsy.2021.818

**Published:** 2021-08-13

**Authors:** S. Ait Bouighoulidne, A. Elgot, F.Z. Laamiri

**Affiliations:** Sciences And Health Technologies, Higher Institute of Health Sciences Hassan First University, settat, Morocco

**Keywords:** COVID-19, drug consumption, lockdown, online survey

## Abstract

**Introduction:**

The emergence of COVID-19 changed the lifestyle of individuals through the appearance of lockdown. This period seems to have potential effect on some health determinants related to behaviors such as drug addiction.

**Objectives:**

Compare the prevalence and behavior of drug consumers before and during the lockdown.

**Methods:**

A cross-sectional study was carried out on 1001 Moroccan addict between April and July 2020, using two international questionnaires: The global drug survey “Special Edition on COVID-19” and the survey on impact of COVID-19 on patients and families.

**Results:**

before lockdown, tobacco use (80.2%), alcohol (70.9%), cannabis (46.3%). During lockdown: tobacco consumption remains unchanged (80.7%), alcohol and cannabis consumption reduced significantly, respectively (39.6%) and (40.8%). Results show also the weaning of hallucinogens and solvents. 76.3% decreased their use, the raisons behind these changes were: 39.8% worried about health issues, 26.6% have less opportunities to consume, 23.7% think that the current life style make it difficult to use, 23.4% think they have less ability to obtain drugs.

**Conclusions:**

The lockdown seems to be an opportunity for addicts to reduce drugs consumption. However, setting up support services with targeted interventions is the best chance to decrease psychological stress and avoid the consequences of this reduction.

